# Different Extraction Approaches for the Analysis of Melatonin from Cabernet Sauvignon and Feteasca Neagra Wines Using a Validated HPLC-FL Method

**DOI:** 10.3390/molecules28062768

**Published:** 2023-03-19

**Authors:** Sandra A. V. Eremia, Camelia Albu, Gabriel L. Radu, Marian Ion

**Affiliations:** 1 Centre of Bioanalysis, National Institute of Research and Development for Biological Sciences–Bucharest, 296 Splaiul Independentei, 060031 Bucharest, Romania; 2 Institute for Research and Development for Viticulture and Wine Making, 2 Valea Mantei, Valea Calugareasca, 107620, Romania

**Keywords:** melatonin, wines, SPE, QuEChERS, DLLME, HPLC-FL, validation, measurement uncertainty, Feteasca Neagra, Cabernet Sauvignon

## Abstract

In recent years, the wine industry has shown a considerable degree of interest in the occurrence of melatonin in wines. Sample pretreatment may be the most important step in trace analysis. Since wine is a complex matrix and melatonin is present in low amounts (ppb), an adequate extraction technique is required. In this study, the effect of several extraction methods, such as solid phase extraction (SPE), Quick, Easy, Cheap, Effective, Rugged, and Safe extraction (QuEChERS), and dispersive liquid–liquid micro-extraction (DLLME) was studied and the variable parameters that can arise throughout the extraction process were optimized to obtain the best results. A high-performance liquid chromatography with fluorescence detector (HPLC-FL) method was adapted and validated, including measurement uncertainty, for the analysis of melatonin in wines and to assess the efficiency of the extraction yield. After comparing the acquired results, the DLLME method was optimized. Extraction recoveries values ranging from 95 to 104% demonstrated that the approach may be successfully applied for the extraction and concentration (enrichment factor of almost eight) of melatonin in wine samples prior to HPLC-FL analysis. The first report of melatonin levels in Feteasca Neagra wines has been made. The data obtained for Cabernet Sauvignon revealed that the final levels of melatonin in the wines are dependent on the winemaking process.

## 1. Introduction

Due to its high concentration of bioactive components with antioxidant characteristics, wine, an alcoholic beverage with potential health benefits, merits special consideration. Melatonin, a naturally occurring indole hormone with a significant antioxidant effect [1], has been found in a variety of foods and drinks, including wine. Melatonin has been shown to be a powerful direct free radical scavenger, and also suppresses prooxidant enzymes, stimulates activity of antioxidant enzymes, and reduces metal-induced toxicity and radical formation due to improving mitochondrial function [2]. In recent years, the presence of melatonin in wines [3] has aroused a significant deal of attention in the wine business due to its possible additive and synergistic effects with other antioxidants [4] that ensure a higher cytoprotective effect against oxidative stress, thus supplementarily supporting the premise that health benefits of the Mediterranean diet are due in part to wine [5]. In addition to their polyphenol content [6] and antioxidant activity, the presence of melatonin in wines should be included when assessing their antioxidant effectiveness, and a trustworthy technique for its determination is required. 

Numerous complex analytical methods were developed for the analysis of melatonin from various matrices (tablets, serum, plant material, milk, etc.), such as: the differential pulse voltammetry method [7], the enzyme-linked immunosorbent assay (ELISA) method [8], the capillary electrochromatography method (CEC) [9], and the methods of HPLC [10,11]. HPLC is the sole trustworthy technology since it has a large capacity for discrimination, permits unambiguous identification, and performs one of the most precise analyses. For the analysis of the wine samples, the following procedures were used: HPLC with diode array detection (HPLC-DAD) [12], HPLC-FL using the native fluorescence of melatonin [3,13,14], and HPLC tandem mass spectrometry (HPLC-MS/MS) [15,16]. Even though the MS detector is more sensitive than the FL detector, HPLC-MS/MS is too complex and costly, making HPLC-FL more cost-effective and suited for assaying melatonin in wines, particularly after sample purification and concentration [17,18].

The initial and perhaps critical stage in qualitative/quantitative analysis is sample pretreatment. Since wine is a complex matrix and melatonin is present in low concentrations (ng mL^−1^) [19], it will be advantageous to use an extraction approach owing to its isolation, interference removal, and concentration capabilities. For the pretreatment of melatonin from wines, various extraction methods were evaluated, including SPE, the most used sample preparation method [3,17,18], as well as the miniaturized form of SPE, microextraction by packed sorbent (MEPS) [13] and ultrasound-dispersed liquid–liquid microextraction (US-DLLME) [12]. In SPE, the selectivity is restricted since the same classic sorbents (chemically functionalized silica, ion-exchange) and mobile phase are routinely used for the extraction and purification of quite distinct analytes from entirely different matrices [20]. Since DLLME employs small quantities of organic solvents, it becomes an attractive sample pretreatment procedure with several benefits, such as speed, simplicity, efficiency, cheap cost, etc. Also, in recent years, rapid, simple, inexpensive, efficient, robust, and secure extraction, QuEChERS, has been used for removing matrices and extracting melatonin from food samples [21,22]. Since the established QuEChERS techniques for the extraction of melatonin from food samples offer several benefits, such as simple operation, minimal consumption of organic reagents, and acceptable recoveries, this approach should also be enhanced for the extraction of melatonin from wine samples.

However, these approaches have various extraction yields and efficiency, and it may be feasible to increase the concentration of melatonin in wine samples by adjusting the extraction settings and optimizing the variables to determine the optimal sampling conditions.

This study aims to investigate the influence of different extraction methods and to optimize extraction process variables (solvent concentration, type of solvents and salts, liquid-to-liquid ratio) to obtain the best results for wine sampling before HPLC-FL analysis of melatonin from wines. Therefore, several extraction techniques, such as SPE, QuEChERS, and DLLME, were evaluated to determine the most efficient approach for separating and concentrating melatonin from wine samples. Using a validated HPLC-FL technique for the qualitative and quantitative study of melatonin in Cabernet Sauvignon and Feteasca Neagra wines, the extraction yield efficiency was examined.

## 2. Results and Discussion

### 2.1. Validation and Uncertainty Estimation of HPLC-FL Method

To achieve the proposed goal, a melatonin analysis method is needed to assess the efficiency of extraction methods and firstly, a previously developed HPLC-FL method [23] was adapted and validated by following some method performance parameters recommended in the guidelines of AOAC [24,25], including selectivity, linearity, limit of detection, precision, accuracy, and robustness. In addition, the uncertainty of the method was assessed [26]. 

Melatonin has native fluorescence and does not require the use of a derivatization reagent when sampling. Therefore, to demonstrate the selectivity of the HPLC-FL method, it is necessary to distinguish between the retention times of melatonin and other native fluorescence-interfering substances that may be present in the wine matrices. By overlaying chromatograms of Cabernet Sauvignon and Feteasca Neagra wines with those of wine samples spiked with melatonin, a difference was observed between the retention times (t_R_) of the specific melatonin peak (23.58 ± 0.02 min) and those of the wines’ matrices compounds. The values for the chromatographic resolution of the retention times obtained between 1–1.5 also confirm the selectivity of the method.

By injecting seven different concentrations of standards in triplicate, the regression curves (A = f(c), A is peak area and C is melatonin concentration), the linear range of the response, and the correlation coefficients (R) were estimated. As can be seen from Table 1, the linearity range of the response was 1.5 decades, namely, the R was higher than 0.9996. The limit of detection (LoD) and limit of quantification (LoQ) were calculated as three times the signal-to-noise ratio and 10, respectively, and the values obtained confirm that the method can be used for the quantitative analysis of melatonin from wine samples. 

The precision of the method was evaluated by estimating the repeatability and the reproducibility of the method (Table 2 and Table 3). In order to assess the injection and the repeatability of the analysis by the same analyst, method, and instrument in one day, the results of five repetitive injections for two different concentrations (2.5 and 5 ng mL^−1^ melatonin standard) were used for calculating the coefficients of variation (CVs). The resulting CVs values for repeatability are below 1% confirming the repeatability of the validation method. For the day-to-day reproducibility of the method, five consecutive melatonin (10 ng mL^−1^) injections prepared every day on three different days for five months were achieved and the CV value obtained is almost 1%.

The accuracy of the method (recovery) was assessed using two wine samples spiked with melatonin, with each sample being analyzed five times. In the case of method recovery, the values obtained were in the range of 97.75–106.65%, proving that the HPLC-FL method for the concentration level used (5–15 ng mL^−1^) was accurate enough to not have any errors, which can significantly affect the analysis results.

To demonstrate the robustness of the method, injections of standard melatonin were performed with minor modification to HPLC operating parameters, such as column temperature (20 °C and 23 °C) and detector cell temperature (24 °C and 25 °C). The results obtained showed that the changes occurring in the values of the retention times or peak areas for melatonin are minor and still allow the compound to be identified using the retention time criterion and do not affect the linearity of the response. 

To calculate the measurement uncertainty, all potential sources influencing the total uncertainty were identified (uncertainty related to the preparation of the calibration solution, uncertainty related to calibration curve, uncertainty related to the precision of measurements) and evaluated for the combination to form a relative total uncertainty. All potential sources of uncertainty were calculated as standard uncertainties and a normal/rectangular/triangular distribution of true values was assumed. 

The uncertainty associated with the preparation of the calibration solution (*u*_prep_) was calculated and a small value was obtained: *u*_prep_ ≈ 0.05. 

The sample preparation has a small influence on the overall measurement uncertainty.

The uncertainty associated with the calibration curve (*u_cal_*) depends on the value of the confidence interval, it was estimated using the regression parameters. and the result calculated was *u_cal_* = 0.0857. 

One of the main factors affecting measurement uncertainty is the uncertainty associated with precision of measurements (*u_precision_*). To calculate the full contribution of the uncertainty associated with precision of measurements, all precision factors were used; *u*_precision_ related to method precision is 1.11. The uncertainty associated with the precision of measurements repeatability has the greatest impact on the melatonin determination. The reason for this lies in the fact that measurement of precision encompasses the entire measurement process from sample preparation to the calibration process, and at the end, the result calculation. Therefore, in order to improve the method, efforts should be focused on reducing the uncertainty of the precision measurement. 

The next step after estimating all uncertainty components expressed as standard uncertainties (*u*) was to calculate the combined standard uncertainty and value of *u*, which is 1.114.

The last step was the calculation of the expanded measurement uncertainty based on the combined standard uncertainty. The extended total uncertainty, U, is a dimension that represents the range of measurement results within which a result falls with a certain degree of confidence. The selection of the coverage factor k (usually 2 or 3) depends on the confidence level (95% or 99%).

Considering a rectangular distribution of the values, a value of U obtained was 2.22. 

The measured result can be expressed as X = x ± U (95% confidence level).

### 2.2. Different Extraction Methods of Melatonin from Wine

In order to achieve the objective of this work, first, the SPE was studied to optimize the extraction procedure of melatonin from wines. Due to the fact that melatonin is a small non-polar and amphiphilic molecule, it was chosen for the extraction of the Bond Elut C18 cartridge, the most hydrophobic and well-known SPE sorbent due to its excessive retention of non-polar compounds. The choice of methanol as the elution solvent was made considering the solubility of melatonin in different solvents [27], in order to remove as many interferences as possible with native fluorescence from the wine matrix. Melatonin is present in low or very low concentration in wine samples, so it needs to be preconcentrated with as high a wine: methanol ratio as possible. However, after monitoring the efficiency of different SPE methods of melatonin-enriched wine samples by HPLC technique, it was concluded that the best ratio is 1:1. These disadvantages of not concentrating and incomplete removal of interferences made us choose another extraction technique used for separation, purification, and concentration. 

QuEChERS was first introduced to remove pesticides from a variety of products, but in recent years it is widely used to purify various compounds from different matrices due to the exploitation of a variety of adsorption fillers. QuEChERS can purify with exceptional matrix interferences elimination, has reasonable extraction capacity, and achieves satisfactory recoveries for melatonin [28]. Since QuEChERS also has several other advantages, we tested this extraction technique when sampling melatonin from wine samples. The QuEChERS method had used a one-step acetonitrile (MeCN) extraction and a salting out of the liquid–liquid partitioning from the wine in the sample with NaCl and MgSO_4_. A dispersive-solid-phase extraction (dispersive-SPE) was performed to remove organic acids, excess water, and other components using a combination of primary secondary amine (PSA) sorbent, Bulk Carbograph, and MgSO_4_. Several liquid-to-liquid wine: MeCN ratios were tested to select the optimal one, and the best results for greatest interference removal were observed using a 1:9 wine: MeCN ratio. After monitoring the repeatability, it was observed that the values of the obtained coefficients of variation were higher than five, proving that the method is not robust enough. 

To further improve the concentration ability and extraction efficiency, the DLLME method for melatonin extraction from wine samples was tested. In general, DLLME is a simple method that offers high recovery values with low solvent consumption, while concentrating on the analyte of interest. Extraction was attempted with MeCN as dispersion solvent, chloroform as extraction solvent, in wine: MeCN: chloroform, 3:3:1, and with NaCl as salt for salting-out effect. Purification and concentration of melatonin was observed as a result of HPLC analyses for identification and quantification. After comparing the results obtained with the three extraction methods, it was decided to continue the study with the DLLME method. This method is the most robust as it satisfactorily removes interferences from the sample matrix, concentrates the analyte of interest so that the sample can be analyzed by HPLC-FL, and gives the best extraction yield (Table 4).

**Figure 1:**
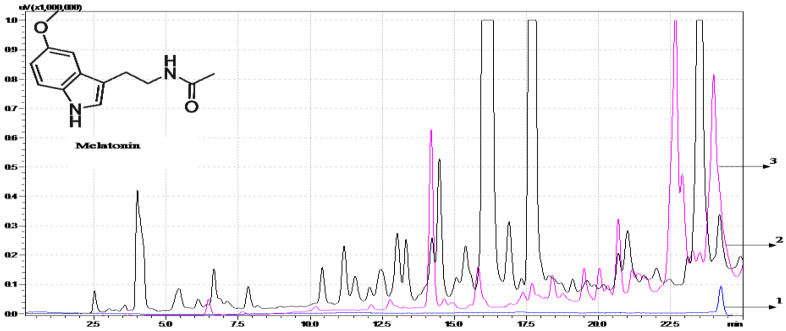
Chromatograms obtained for melatonin—standard and wine samples (blue—standard, black—wine sample, pink—wine sample after DLLME, 1,2,3—melatonin peaks) by HPLC-FL (λ_ex_ = 285 nm, λ_em_ = 340 nm).

DLLME can be customized for a specific purpose and incorporate other sample preparation techniques. Furthermore, the parameters influencing DLLME (the selected salt for the salting-out effect, the amount of salt added to the sample, the ratio of wine:dispersion solvent:extraction solvent) were optimized. To evaluate the increase in ionic strength of the sample solution, NaCl, Na acetate, and MgSO_4_ were added as potential salting-out agents. The results obtained by HPLC-FL showed that NaCl is a suitable salting-out agent and is used for further experiments. The amount of salt added should be enough to saturate the sample, so the optimization was performed with different amounts of NaCl (0.5 g, 1 g, and 1.5 g). The addition of 1 g NaCl provided a well-resolved chromatogram with the highest peak area for melatonin and was thus chosen as the optimal amount of the salt. In order to obtain a high enrichment factor, defined as the ratio of the melatonin concentration in the sedimented phase to the initial concentration of melatonin in the wine sample, it is important to optimize the solvent volumes used. The effect of the ratio of wine sample, dispersant, and extraction solvent on extraction efficiency was studied. During the optimization of the extraction procedure, many different sample to solvent ratios were tested and the 11.(3):1:1 ratio had the largest enrichment factor (nearly 8) (Figure 1). 

Based on the values of extraction recovery obtained (between 95–104%), comparable to the results obtained by Viegas [14], we can conclude that the proposed DLLME method is the most appropriate and can be successfully applied to extraction and concentration of melatonin in wine samples, before analysis by HPLC-FL. 

### 2.3. DLLME-HPLC-FL Method Application in Wine Samples

The proposed DLLME extraction followed by the validated HPLC-FL method was applied to two Romanian wine varieties, Cabernet Sauvignon and Feteasca Neagra, confirming the efficiency of these procedures. The wine samples were obtained by two different maceration fermentation processes, described in Section 3. Materials and Methods. The melatonin levels in two Romanian red wines are presented in Table 5. 

Feteasca Neagra is an old autochthonous grape variety from Romania, from which red wines are made. The variety is only grown in Romania and Moldova and for the first time in this study, the presence of melatonin in this wine was reported. The Cabernet Sauvignon values obtained were comparable to those from Portuguese wines by HPLC-FL, 2.85–3.06 ng mL^−1^ [14], 0.32 ng mL^−1^ by CEC methods [9], or 14.2 ng mL^−1^ analyzed with HPLC-MS/MS [17].

Comparing the values found in wines made by the pumping over maceration process with those found in wines made by the punch down maceration process for both Feteasca Neagra and Cabernet Sauvignon, it was observed that higher melatonin concentrations were obtained by the first technique. The final amounts of melatonin from wines depend on the grape variety, the geographic regions of the vineyards, the winemaking process, and the analytical method used for quantification.

## 3. Materials and Methods

### 3.1. Reagents

Melatonin (Sigma, Steinheim, Germany, M5250) stock solution, 1 mg mL^−1^, was prepared in ethanol (Riedel-de Haen, Seelze, Germany), protected from light and stored at 4 °C. All other used reagents, MeCN (Riedel-de Haen), chloroform (Sigma, 366919), methanol (Riedel-de Haen,), NaCl (Sigma, S7653), MgSO_4_ (Sigma, M7506), sodium acetate (Sigma, S8750), and acetic acid (Sigma, A6283), were of analytical or chromatographic purity. The ultra-pure water was obtained with an Elix 3 (Millipore, Darmstad, Germany) system. 

### 3.2. Wine Sampling 

Two different standardized wine samples were tested. The wines were obtained from the noble grape varieties Cabernet Sauvignon and Feteasca Neagra from the Romanian geographic region of Valea Calugareasca.

The wines were obtained through the traditional process of maceration for both grape varieties using punch, the process of breaking the cap and immersing in the must, 2 times a day for 7 days, as well as pumping over, the process of getting the juice from the soil of the fermentation tank and pumping it over the cap, once a day for 7 days. After applying an extraction technique of melatonin from wines, samples were filtered in HPLC vials and injected into the HPLC system.

### 3.3. SPE Method

To remove as many interferences from matrices as possible, the SPE was prepared using Bond Elut C18 cartridges (500 mg and 3 mL, Agilent, Santa Clara, CA, USA) and methanol as the elution solvent. First, the SPE cartridges were activated with 2 mL methanol and then equilibrated with 2 mL water. The cartridge was loaded with 0.75 mL wine sample which was first centrifuged at 10,000 rpm for 5 min and the cartridge was washed with 0.75 mL methanol. The eluted samples containing the compound of interest were analyzed using an HPLC method. 

### 3.4. QuEChERS Method

A total of 1 mL of wine sample was placed in a centrifuge tube with 9 mL MeCN and mixed by vortexing at 1200 rpm for 2 min. A mixture of extraction salts (4 g MgSO_4_ + 1 g NaCl), was added to the samples being vortexed at 1200 rpm for 4 min and centrifuged at 10,000 rpm for 5 min. A total of 5 mL of the supernatant was transferred to a 15 mL dispersive SPE tube, High Pigment (Agilent, Bond Elut, 5982-5356) containing a salt mixture (149.7 mg PSA, 44.9 mg Bulk Carbograph, 855.4 mg MgSO_4_). The SPE tube was vortexed for 4 min and centrifuged at 10,000 rpm for 5 min. The supernatant was then transferred to HPLC vials for HPLC analysis. 

### 3.5. DLLME Method

DLLME is an extraction technique in which the fine droplets of an extraction solvent are dispersed in an aqueous sample, in this case, wine samples. Our study used MeCN as the dispersing solvent (completely soluble in the water phase) and chloroform as the extraction solvent (soluble in the dispersing solvent while its solubility in water must be very low). From the supernatant of the centrifuged wine sample (5000 rpm for 5 min), 8.5 mL were transferred to a suitable tube and 1.5 mL chloroform: MeCN, 1:1 *v*/*v* was added by rapid pipetting. The solution was mixed by vortexing at 1200 rpm for 10 min. A total of 1 g NaCl salt was added to the sample for the salting-out effect, a purification technique that exploits the reduced solubility of specific molecules in a very high ionic strength solution and vortexed for 5 min. After centrifuging the samples at 5000 rpm for 5 min, the analyte of interest is found in the lower phase, collected, filtered into HPLC vials, and injected into the HPLC system. 

### 3.6. HPLC Analysis

Melatonin analysis was performed using an HPLC Shimadzu (Kyoto, Japan) consisting of a SIL-20AC autosampler, two LC-20AD pumps, a DGU-20A degasser, a CTO-20A column oven, an RF-20A XS fluorescence detector set at λex = 285 nm and a λem = 340 nm, and LC Solution software. The fluorescence detector parameters were: 1.5 sec response, Cell Temperature Control at 25 °C, Gain×4, medium sensitivity. The Cell Temperature was chosen after comparing the results (peak intensity and peak area values) obtained in the case of melatonin assays using detector cell temperatures of 20 °C, 25 °C, and 30 °C.

The identification and quantification of melatonin by HPLC-FL was performed on a Kromasil, Sweden, 100-5-C18 4.6 × 250 mm column and an elution gradient of mobile phase (methanol, solvent A, and 1% acetic acid, solvent B, 0–3 min 10–15% solvent B, 3–25 min 15–50% solvent B, 25–30 min 10% solvent B) with 1 mL min^−1^ flow rate. The analyses were performed over 30 min, at a temperature column of 20 °C and an injection volume of 20 µL. Samples were filtered before an injection using a 25 mm, 0.2 µm Syringe-Driven Filter Unit (PVDF, Agilent). The column was equilibrated for 30 min before injections.

The results obtained after triplicate injection of each sample are expressed as the mean ± standard error.

### 3.7. Measurement Uncertainty

The uncertainty associated with the preparation of the calibration solution, *u_prep_*, is a compound uncertainty and consists of only three sources of uncertainty: uncertainty of the weighing, *u*(*m_st_*) (weighing 1 ± 0.1 mg of the specified standard), uncertainty of the purity, *u*(*P*) (melatonin purity (*P*) is given as ≥ 98%), and uncertainty of the pipetting, *u*(*V_P_*_1_–*_P_*_3_) (the manufacturer tolerance for the 10 µL, 1 mL, and 5 mL pipette is 1 ± 0.009, 1 ± 0.0026, and 5 ± 0.03 mL, respectively, at 20 °C). *u_prep_* was calculated using Equation (1), where the *u*(*m_st_*) is 0.05 mg, the *u*(*P*) is 0.005 mg/g, and the *u*(*V_P_*_1–*P*3_) are *u*(*V_P_*_1_) = 0.0036 mL, *u*(*V_P_*_2_) = 0.001 mL, *u*(*V_P_*_3_) = 0.012 mL.

(1) $$u_{prep}=\sqrt{{\left(\frac{u\left(m_{st}\right)}{m_{st}}\right)}^{2}+{\left(\frac{u\left(V_{P1}\right)}{V_{P1}}\right)}^{2}+{\left(\frac{u\left(V_{P2}\right)}{V_{P2}}\right)}^{2}+{\left(\frac{u\left(V_{P3}\right)}{V_{P3}}\right)}^{2}+{\left(\frac{u\left(P\right)}{P}\right)}^{2}}$$

The uncertainty associated with the calibration curve, *u_cal_*, was estimated using the regression parameters (*u_x_*) in Equation (2).

(2) $$u_{cal}=\sqrt{\sum_{x=1}^{n}u_{x}^{2}}$$

The precision of measurements (repeatability and reproducibility, intermediate precision) was quantified in terms of the standard deviation or CVs of the measured value.

The next step after estimating all uncertainty components expressed as standard uncertainties (*u*) is to calculate the combined standard uncertainty using the following expression, Equation (3):

(3) $$u=\sqrt{\sum_{i=1}^{n}u_{i}^{2}}$$

where *u*_i_ are *u_prep_, u*_cal_, and *u_precision_*, respectively.

Considering a rectangular distribution of the values and a confidence interval of 95% with a coverage factor *k* = 2, a value of *U* was obtained from Equation (4).

(4) U=k*u=2*1.11=2.22

## 4. Conclusions

In this work, after studies to obtain the best results in terms of the extraction and concentration of melatonin before HPLC-FL analysis of wines, it can be concluded that the method proposed by DLLME is the most suitable. To achieve the aim of this study, an HPLC-FL method was adapted and validated including the measurement uncertainty term for the analysis of melatonin from wine samples. For the first time, Feteasca Neagra wines have been analyzed using the extraction method proposed by DLLME together with the validated HPLC-FL method and melatonin content data reported. Also, the data obtained for Cabernet Sauvignon, consistent with those previously reported in the literature, confirmed that the final levels of melatonin in the wines depend on the winemaking process and that the DLLME-HPLC-FL method is an optimal analytical tool to analyze this target analyte in wine tasting.

## Figures and Tables

**Table 1:** Some performance characteristics of the HPLC-FL method used for melatonin analysis of wine samples.

Compound	λex nm	λem nm	t_R_ min.	The Linear Regression Equation ng mL^−1^	R	Linearity of Response ng mL^−1^	LoD ng mL^−1^	LoQ ng mL^−1^
Melatonin	285	340	23.58	A = 219403.4 × C − 248.5414	0.9996	1–25	0.01	0.05

λ_ex_—excitation wavelength; λ_em_—emission wavelength.

**Table 2:** The experimental results obtained to demonstrate the repeatability of the HPLC-FL method.

Analyte	Date	Conc. ng mL^−1^	Peak Areas	CV	t_R_	CV
Melatonin	22.06	2.5	1,183,246	0.23%	23.61	0.09%
			1,184,829		23.56	
			1,179,878		23.58	
			1,183,979		23.58	
			1,178,708		23.60	
		5	1,420,635	0.97%	23.59	0.11%
			1,431,014		23.59	
			1,438,296		23.60	
			1,456,557		23.62	
			1,446,875		23.65	

Conc.—concentration.

**Table 3:** The experimental results obtained to demonstrate the reproducibility of the HPLC-FL method.

Analyte	Conc. ng mL^−1^	Date	Conc.ng mL^−1^	Conc. ng mL^−1^	CV
Melatonin	10	24.06	10.14	9.98 ± 0.09	0.97%
			9.99		
			10.04		
			10.05		
			9.89		
		29.07	9.95		
			10.09		
			10.12		
			9.92		
			9.85		
		06.10	9.97		
			10.06		
			9.89		
			9.83		
			10.02		

**Table 4:** Extraction yields using different extraction approaches for the analysis of melatonin from wine.

Extraction Method	Wine Concentration ng mL^−1^	Conc. Melatonin Added ng mL^−1^	Conc. Wine + Melatonin Obtained ng mL^−1^	Extraction Yield (%)
SPE	1.25	1	1.10 ± 0.02	49
		10	6.52 ± 0.04	58
		25	16.27 ± 0.06	62
QuEChERS	1.25	1	1.55 ± 0.01	69
		10	9.00 ± 0.08	80
		25	20.47 ± 0.1	78
DLLME	1.25	1	1.96 ± 0.03	87
		10	10.24 ± 0.08	91
		25	24.41 ± 0.12	93

**Table 5:** The melatonin values obtained by the proposed DLLME-HPLC-FL method from two red wines.

Wine Varieties	Melatonin ng mL^−1^	
	*Punchdown* Method	*Pumping Over* Method
Feteasca Neagra	0.74 ± 0.06	1.09 ± 0.09
Cabernet Sauvignon	0.84 ± 0.06	1.36 ± 0.05

## Data Availability

The authors confirm that the data supporting the findings of this study are available within the article.
